# Casein and Whey Protein in the Breast Milk Ratio: Could It Promote Protein Metabolism Enhancement in Physically Active Adults?

**DOI:** 10.3390/nu13072153

**Published:** 2021-06-23

**Authors:** Bryan S. Martinez Galan, Flavia Giolo De Carvalho, Simone C. S. Carvalho, Camila F. Cunha Brandao, Sara I. Morhy Terrazas, Gabriela Ferreira Abud, Monica S. S. Meirelles, Simone Sakagute, Gabriela Ueta Ortiz, Julio S. Marchini, Juan C. Aristizabal, Ellen Cristini de Freitas

**Affiliations:** 1 Department of Food and Nutrition, School of Pharmaceutical Sciences of Araraquara, State University of Sao Paulo–FCFAR/UNESP, Araraquara 14800-903, Brazil; bryan.mg@hotmail.com (B.S.M.G.); saraterrazas@yahoo.com.br (S.I.M.T.); gabrielaabud.nutri@hotmail.com (G.F.A.); 2 School of Physical Education and Sports of Ribeirao Preto, Laboratory of Exercise Physiology and Metabolism, University of Sao Paulo (EEFERP-USP), Ribeirao Preto 14040-907, Brazil; flaviagiolo@gmail.com (F.G.D.C.); monicassmeirelles@gmail.com (M.S.S.M.); simone.sakagute.tavares@usp.br (S.S.); 3 Department of Genetics, Ribeirao Preto Medical School, University of Sao Paulo, Ribeirao Preto 14049-900, Brazil; simonedacostaesilva@gmail.com; 4 Internal Medicine Department, Ribeirao Preto Medical School, University of Sao Paulo, Ribeirao Preto 14049-900, Brazil; camila.brandao@uemg.br (C.F.C.B.); jsmarchi@fmrp.usp.br (J.S.M.); 5 Faculty of Physical Education, State University of Minas Gerais, Divinopolis 35501-170, Brazil; 6 Department of Health Sciences, Ribeirao Preto Medical School, University of Sao Paulo, Ribeirao Preto 14049-900, Brazil; gabrielaueortiz@gmail.com; 7 Nutrition and Dietetics School, Physiology and Biochemistry Research Group, Universidad de Antioquia, Medellin 050010, Colombia; juan.aristizabal@udea.edu.co

**Keywords:** amino acids profile, DOMS, resistance exercise, nitrogen balance

## Abstract

Due to the utilization of milk proteins such as whey protein (WP) and casein as sports nutrition ergogenic aids, the present study investigated the effects of the association of WP and casein in a ratio of 80:20, a similar ratio of human breast milk, on blood branched-chain amino acid (BCAA) profiles, markers of protein metabolism and delayed onset muscle soreness (DOMS), after a single bout of resistance exercise. A double-blind, crossover and acute study was carried out with ten men (age 29 ± 8 years; BMI: 25.4 ± 2.9 kg/m^2^; 77 ± 12 kg; 1.74 ± 0.09 m); each one consumed the following supplements randomly, one per session: WP, CAS (casein), WP/CAS (80% WP/20% CAS), CAS/WP (80% CAS/20% WP) and PLA (placebo). They were also subjected to the following evaluations: the one repetition maximum (1RM) test; resistance training session; blood extraction during each session to determine the BCAA profile; two food records; 3-day evaluation of DOMS (24 h, 48 h and 72 h) and nitrogen balance in each treatment. The intervention resulted in similar nitrogen urinary, creatinine and urea plasma levels and showed a positive nitrogen balance in all the trials. Regarding the BCAAs, the peak occurred at 60 min post-ingestion and remained higher until 120 min for WP, WP/CAS and CAS/WP. The DOMS was significantly lower for WP, WP/CAS and CAS/WP compared to the CAS and PLA treatments. There were no advantages in the association of WP and CAS in the BCAAs profile when compared to WP itself, but it induced a lower DOMS compared to CAS and PLA (Clinical Trial registration number: clinicaltrials.gov, NCT04648384).

## 1. Introduction

The proteins of human breast milk are the most important endocrine signaling system that promotes neonatal growth by increasing the release of insulin, insulin-like growth factor-1 (IGF-1), and the leucine-mediated mammalian target of rapamycin complex 1-(mTORC1) signaling of pancreatic β-cells [1,2]. Remarkably, the branched-chain amino acids (BCAAs) leucine, isoleucine, and valine are involved in the growth-promoting effects of milk, protein biosynthesis, and metabolism, because they physiologically stimulate insulin secretion [3]. The amount of leucine per gram of milk protein (~1.2 g/100 mL of milk) seems to be the most important factor to induce leucine-mediated growth and muscle protein synthesis due to its major role in mTORC1 activation [1].

In this context, milk proteins such as whey protein (WP) and casein have been used as sports nutrition ergogenic aids for muscle protein synthesis (MPS), muscle mass gain, and post-exercise recovery. The effects of the association of exercise and protein intake in the skeletal muscle protein synthesis is well-stablished [4]. To maximize the training results and maintain a positive nitrogen balance, it is necessary to have an adequate daily protein consumption between 1.6 and 2.2 g per kilogram of body weight, including milk, meat, or other sources of animal and vegetarian proteins, as well as protein supplements when necessary [5,6]. 

Additionally, the type of protein consumed can affect the body protein anabolism differently due to the different characteristics of proteins, such as biological value, absorption speed, amino acid composition, ability to reach the nitrogen and amino acid requirements [7], hormonal responses, or antioxidant effects [8,9,10,11]. According to Jäger R et al. [11], WP and casein contain the highest bioavailability of amino acids. Both supplements are protein substrates derived from milk; however, they have different chemical characteristics that may interfere in the final response to muscle mass gain [8,12,13,14]. 

Casein is slowly released from the stomach to the gut; however, it induces a prolonged release of dietary amino acids, while WP is a soluble protein and fast-digested protein and promotes a faster, but transient, plasma appearance of dietary amino acids compared to casein [15]. When analyzing the amino acid content, WP had a higher content of branched-chain amino acids than casein, highlighting that leucine is the highest one. Previous studies have shown that, beyond amino acid composition, the kinetic profile of protein absorption can modulate protein synthesis. Walrand et al. [16] showed that 15 g of WP compared to 30 g of casein over 10 days induces similar muscle protein synthesis rates in healthy elderly individuals. Therefore, the amino acid content appears to influence MPS more than the amount of protein consumed. 

Considering the benefits of these milk proteins, it is known that, during the initial period of each mammalian’s life, milk is the only food source that fulfills all nutritional requirements [17]. In the same way, human breast milk (HBM) is considered a gold standard protein and gold standard in infant nutrition due to this high biological value, as it contains essential amino acids and thousands of different bioactive molecules that can contribute to protection during the initial stage of life against infection and inflammation and enhance immune maturation, organ development, and healthy microbial colonization [18,19,20]. Due to the differences between WP and casein, and considering breast milk as a gold standard protein that contains around a 80:20 ratio of whey protein to casein [21], would the association of WP and casein in proportions that are similar to human breast milk result in a higher peak and longer permanence of plasma-circulating amino acids and, consequently, improve muscle recovery in physically active individuals? 

Previous studies have suggested that the 80:20 ratio of fast and slowly digested proteins provides greater bioavailability and faster gastric emptying, while cow’s milk has proportions contrary to those described for breast milk, containing a higher proportion of slowly digested proteins, which could influence the protein metabolism function [7,11,22,23,24,25]. 

However, the effects of these proteins in the plasma BCAA levels have not been investigated yet, as well as their levels post-supplement intake during exercise recovery.

Amino acids undergo tightly controlled homeostatic regulation, and their concentrations in blood reflect the interaction between dietary intake, endogenous synthesis, and catabolic and anabolic processes [26,27]. Exercise induces adaptations in amino acid metabolisms, and after intense physical exercise events, a decrease in the total circulating amino acid concentration is shown [28,29,30] that could represent the energy expenditure process being executed.

Due to the importance of a rich amino acid environment, mainly with the high availability of BCAA, to activate protein synthesis pathways such as mTOR (mammalian target of rapamycin) [25], mainly post-exercise leucine-enriched essential amino acids enhance myofibrillar protein synthesis and are suggested to be important for muscle recovery, as described by Waskiw-Fored et al. [31].

The present study sought to investigate the effects of the combination of whey protein and casein in the ratio of 80:20 (“whey protein:casein” or “casein:whey protein”) as a basis for the proportions of fast absorption and slow absorption proteins in the ratios of HBM, which is a gold standard for protein in quality and nutrient contents [21], which could provide changes in the amino acid concentrations of branched-chain amino acids in plasma and cause changes in the bioavailability on the peak and the period of permanence of branched-chain amino acids in the blood circulation, final metabolites of protein metabolism, and delayed onset muscle soreness (DOMS) after a single bout of fasting or a resistance exercise session.

## 2. Materials and Methods

### 2.1. Study Design and Subjects

This is an acute, randomized, crossover, and double-blind trial. Ten healthy males were recruited (age 29 ± 2 years old, BMI 25 ± 3 kg/m^2^, weight 77 ± 12 kg, height 1.74 ± 0.09 m). The exclusion criteria were smokers; muscle injuries; use of drugs related to cardiovascular diseases, diabetes mellitus, and thyroid; executing a resistance training program of less than 3 times per week; or lactose intolerance. Participants trained on their own according to their own goals, being amateur participants with experience in weight training exercises. The volunteers were submitted to sessions of familiarization with a leg press exercise and determination of maximum strength (1RM) to carry out the training protocol and were instructed to not perform physical activity for 48 h before testing. The order of supplementation intake was randomly assigned to the participants: (1) whey protein (WP), (2) casein (CAS), (3) whey protein 80% and casein 20% (WP/CAS), (4) casein 80% and whey protein 20% (CAS/WP), and (5) placebo (PLA). After randomization, the volunteers underwent one session of strength exercise; the supplementation protocol and biological sample collection were performed, as well as one-week washout between each supplement. All participants underwent five supplementation protocols in random order. 

Participants were requested to attend five visits to the Laboratory of Exercise Physiology and Metabolism (LAFEM) of the School of Physical Education and Sport of Ribeirao Preto, University of Sao Paulo (EEFERP USP), where blood samples were collected at the baseline period (PRE), immediately after the exercise–supplementation protocol, and every 60 min for the following five hours post the exercise session and protein supplementation. All the baseline blood collections were performed; participants were instructed to attend the lab early in the morning, between 5:00 and 7:00 a.m., respecting the 10 h of fasting required. In addition, the volunteers were asked to complete food records pre and within 24 h after the training protocol (POST), as well as urine collection during 24 h after the supplementation protocol. The subjects were instructed to keep their diet pattern during all the interventions. 

The study started with 18 participants; however, only 10 completed the intervention and all evaluations proposed in the study. Eight participants were excluded due to personal problems, incomplete protocols, change of residence, and limited hours to participate. The Ethical Committee of the School of Physical Education and Sport of Ribeirao Preto, University of Sao Paulo (protocol number: 64743116.5.0000.5659) approved the study. All subjects gave free written consent prior to participation.

For this experiment, 10 individuals were calculated as sufficient to evaluate the outcomes in the plasma amino acid concentration, which was the primary variable of interest, with a mean difference of 3.0 (SD 2.83), a significance level of 0.05, and statistical power of 80%. The studies conducted by Fobre et al. [32] and Nakayama et al. [33] with similar sample sizes demonstrated that the consumption of higher fast protein increased the plasma amino acid concentration in healthy men and were also considered as references to determine the sample size.

**Figure 1:**
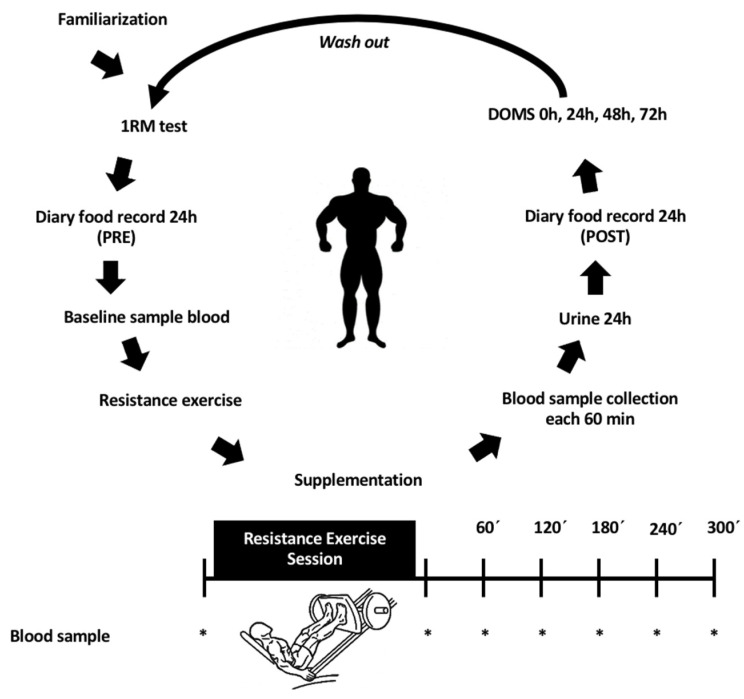
Protocol used during the experiment period. An acute, randomized, and double-blind trial. 1RM: One (1) maximum repetition; DOMS: Delayed onset muscle soreness assessment. Washout: a period of at least of one week.

Figure 1 summarizes the study protocol.

### 2.2. Supplementation Protocol

Supplementation of casein or whey protein was performed according to the double-blind and crossover model, considering the dose of 20 g in the different proportions as described: (1) WP—composed of 100% whey protein, CAS—composed of 100% casein, WP/CAS—blend composed of 80% whey protein and 20% casein, CAS/WP—blend composed of 80% casein and 20% whey protein, and (5) PLA—composed of maltodextrin. Amino acid composition of each protein supplement (g/100 g) is shown in Table 1. The supplements were packaged in individual sachets (provided by the NUTRATEC^®^ company, Ribeirao Preto, Brazil), which were diluted in 250 mL of water and given to the participants to consume immediately after the exercise session. After a minimum period of one week of washout, a new supplementation protocol was started with a different supplement, which was determined by an aleatory random draw performed by a researcher from our lab that was not related specifically to this research project, in order to guarantee the blindness of the protocol. The protocol was repeated until the participants went through all five different types of supplements.

### 2.3. Exercise Intervention

The volunteers participated in three sessions of familiarization with the leg press exercise until the maximum strength measure (one-repetition maximum test—1RM) was stable, with an interval of 48 h between sessions. At the beginning of the familiarization sessions, the volunteers did a five-minute warm-up on the stationary bike [34]; subsequently, they performed a specific warm-up of 5 to 10 repetitions with a light load (40% to 60% of the perception of the 1RM). After a 1-minute interval, a new series of 3–5 repetitions was performed with a moderate load (60% to 80% of the 1RM). The exercise protocol consisted of 10 sets of 8–10 repetitions with a 2-min interval between each set, considering a load of 85% of the 1RM; adapted from Tipton et al. [25], the load was controlled until fatigue; when necessary, there was an increase or decrease from 1% to 5% of the total loading charge. Rating of perceived exertion (RPE) was evaluated using the scale described by Foster et al. [35,36], and the training impulse (TRIMP) for the global training load was calculated by multiplying the session’s RPE score (intensity) by the total session duration in minutes (volume), including warm-up, calm down, and active pauses between efforts [37,38]. The expected metabolic requirements of the strength exercise sessions were calculated based on the Compendium of Physical Activities [39] with assumptions (6.0 MET per session) for the resistance exercise and using the equation for the caloric cost of physical activity [39,40]. The described procedure was performed in the Cineanthropometry and Human Performance Laboratory-LaCiDH of the School of Physical Education and Sport of Ribeirao Preto, University of Sao Paulo (EEFERP USP).

### 2.4. Delayed Onset Muscle Soreness (DOMS) Assessment

DOMS in the movements of sitting and going down the stairs was assessed using a visual scale that consisted of a 100-mm line where, at the one extreme, it represented “no pain” and, at the other extreme, “a lot of pain” [41]. The visual scale values were measured at 0, 24 h, 48 h, and 72 h after the exercise session and supplementation protocol. The participants filled out an individual format; during the 3 days after the exercise protocol, the researchers reminded the participants to draw a line or a trace between those 100 mm that would represent their pain at the time of evaluation.

### 2.5. Biochemical Analysis

Blood samples were collected in 4-mL tubes with heparin (anticoagulant) during each experimental trial with all participants after an overnight fasting of 10 h. Blood samples were collected at the baseline period (PRE), immediately after the exercise and supplementation session, and every 60 min for a period of five hours after each resistance exercise session (0 h, 1 h, 2 h, 3 h, and 5 h). The volunteers performed the urine collection during the 24 h after supplementation and the acute physical exercise session for later evaluation of the protein metabolism.

### 2.6. Protein Metabolism Markers

#### 2.6.1. Amino Acid Quantification

Plasma amino acids levels were analyzed by High-Performance Liquid Chromatography (HPLC, Shimadzu, Kioto, Japan, model LC 10AD) by the methods described by Deyl [42] and Padovan et al. [43] using the amino acids L-Valine, L-Isoleucine, and L-Leucine as analytical standards (Sigma-Aldrich^®^, St. Louis, MO, USA; 98% purity for HPLC assays). 

Samples of plasma (20 µL) were mixed with methanol for protein extraction; then, methanol was evaporated in the Savant Speed-Vac (Thermo Scientific, model AES 2010, Waltham, MA, USA) for 25 min. The protein fraction result was resuspended in phase A solution (40-mmol/L sodium phosphate solution, pH 6.93, containing methanol (2% *v*/*v*), acetonitrile (2% *v*/*v*), and tetrahydrofuran (2% *v*/*v*)) vortexed for 30 s; filtered through a 0.45-µm membrane; and 40 μL of the reaction product was injected into the HPLC. 

A C18 silica column was used to separate the amino acids according to molecular weight and polarity, and the BCAA were detected by the fluorescence detector Shimadzu model RF535 in 335-mm excitation and 455-mm emission.

The concentrations of each BCAA were determined using the equation obtained in the linear calibration curves, considering the peak area for each analyte in the following concentrations: Valine (0, 625, 1250, 1875, and 2500 µmol/L); Leucine (0, 625, 1250, 1875, and 2500 µmol/L); and Isoleucine (0, 625, 1250, 1875, and 2500 µmol/L), according to the following equation: $AC=\left(\frac{PAA}{PAS}\right)/100\times 2.5$, where AC is the amino acid concentration, PAA is the amino acid peak area obtained, and PAS is the standard amino acid peak area obtained. The samples were analyzed in duplicates for precision and accuracy [42]. 

#### 2.6.2. Creatinine and Urea

Plasma measurements of creatinine and urea were performed using the specific kits of Creatinine and Urea CE kits (Labtestdiagnóstica^®^, Lagoa Santa, Brazil), with a colorimetric reaction in an absorbance spectrophotometer.

#### 2.6.3. Urinary Nitrogen

Each volunteer received plastic flasks with a capacity of 2 L, and were duly instructed in relation to the collection procedure and the correct storage of the sample until it was handed over to the researcher for analysis. After collection, 14-mL aliquots were prepared in falcon tubes and stored in a freezer at −80 °C until the moment of analysis.

Samples of 20 μL of 24-h urine collection, in duplicate, were diluted in 1000 μL of distilled water and, after, were injected by manual syringe injector into the furnace in a stream of oxygen at temperature of combustion of 1100 °C; the mean of 10 injections of each sample were used to determine the total urinary nitrogen by the pyro-chemiluminescence method, [44]. The following equation was used to calculate the nitrogen balance (NB) [45]:

NB = (PTN 24 h(g)/(6.25)) − Urinary Nitrogen 24 h (g) + 4 (g)

#### 2.6.4. Nutritional Assessment

All subjects were instructed to maintain their usual diet while participating in the study. Food records were requested in the previous period (PRE), 24 h before testing, and in the 24 h following the exercise session (POST) for each supplement. Participants were trained to properly fill a food diary according to the protocol [46]. For the nutritional assessment, Dietpro^®^ professional software, version 5i was used to calculate the kcal and macronutrients intake.

### 2.7. Statistical Analysis

Descriptive statistics consisted of the mean and mean standard error (mean ± SEM). Data normality was verified by the Shapiro–Wilk test. Then, an ANOVA one-way or two-way repeated measures test was performed, using the GLM module of SPSS (general linear model). In cases of group*time interactions, the post hoc Sidak was applied. Statistical significance was accepted at a level of *p* < 0.05. All analyses were performed with SPSS Statistics™ 20 software (IBM Corporation, Armonk, NY, USA). 

## 3. Results

To test whether the supplement would increase branched-chain amino acids peak and period of permanence, we subjected the participants to 5 h of blood collection post each treatment. According to the observed plasma amino acid concentrations, there was an interaction between the time of evaluation and supplements group in the valine (*p* = 0.003), isoleucine (*p* < 0.001), and leucine (*p* < 0.001) concentrations. The differences were found when comparing the mean values between the protein and placebo supplements after 60 and 120 min post-treatments (*p* < 0.05). In addition, higher concentrations of isoleucine and leucine were observed in the WP/CAS and WP treatments when compared to CAS (*p* < 0.05), as shown in Table 2.

In general, the peak of the BCAA concentration was reached in the same time point, according to each supplement, highlighting that the CAS/WP and WP/CAS blends showed similar leucine and isoleucine concentrations as WP, which reached peak levels at 60 min post-treatment, while CAS reached the peak only at 120 min post-intake (Table 2).

To further determine the period of permanence, the concentrations of the BCAA in each time point were analyzed. It was observed that peak levels of leucine and isoleucine were reached at 60 min post-treatment and maintained higher than the Rest concentrations until 120 min, and after that, at 180 min, the concentrations of leucine and isoleucine decreased and reached similar concentrations to the Rest time point in the WP, WP/CAS, and CAS/WP treatments. The CAS treatment reached the peak of leucine, isoleucine, and valine only at 120 min post-treatment, and after that, in the 180-min time point, the concentrations decreased and reached similar values obtained in the Rest time point. 

When analyzing valine, the peak levels were reached at the 60-min time point for the WP, WP/CAS, and CAS/WP treatments; however, they were maintained higher than the Rest time point concentrations at 120 min only for the WP treatment. Valine concentrations were similar to Rest at the 120-min time point, except for the WP treatment, which showed valine concentrations similar to Rest only at the 180-min time point, as shown in Table 2. 

Regarding PLA treatment, no changes were observed in the isoleucine and valine concentrations when comparing all time points. Interestingly, it was observed that the concentration of leucine in the PLA treatment was lower at 60 min post-treatment than the Rest time point, and it was maintained lower at 120 min post-treatment. However, the leucine concentration increased after 180 min post-intake and was similar to the Rest concentration time point. Taken together, our results suggest that the WP/CAS and CAS/WP blends promoted higher amino acid concentrations, and they were similar to the WP treatment when compared to the CAS and PLA treatments. However, a longer period of permanence was observed only for leucine and isoleucine concentrations in the WP, WP/CAS, and CAS/WP treatments. The plasma concentration of each branched-chain amino acid (Valine, Isoleucine, and Leucine) for each treatment is shown in Supplementary Figure S1. 

To investigate whether the longer period of permanent amino acids after resistance training would improve the performance, as well as muscle soreness, a training load, 1RM test (before each treatment), and a 3-day assessment of DOMS were applied. Table 3 shows the results of the exercise performance during each training session according to the supplement consumed. No changes were observed in the 1RM test load, the leg press load, and the global training load index (TRIMP RPE and TRIMP Vol). It is important to point out that the exercise protocol load was between 80–85% of the 1RM and the reported RPE and was associated with the high intensity of the effort performed (~85% of the 1RM), which showed mean RPE values between 7.9 and 8.3 that were considered “very difficult” to “maximum” effort classifications, according to references [35,36]. All participants concluded each set between 23 to 24 s and completed the ten series of 10 repetitions.

**Figure 2:**
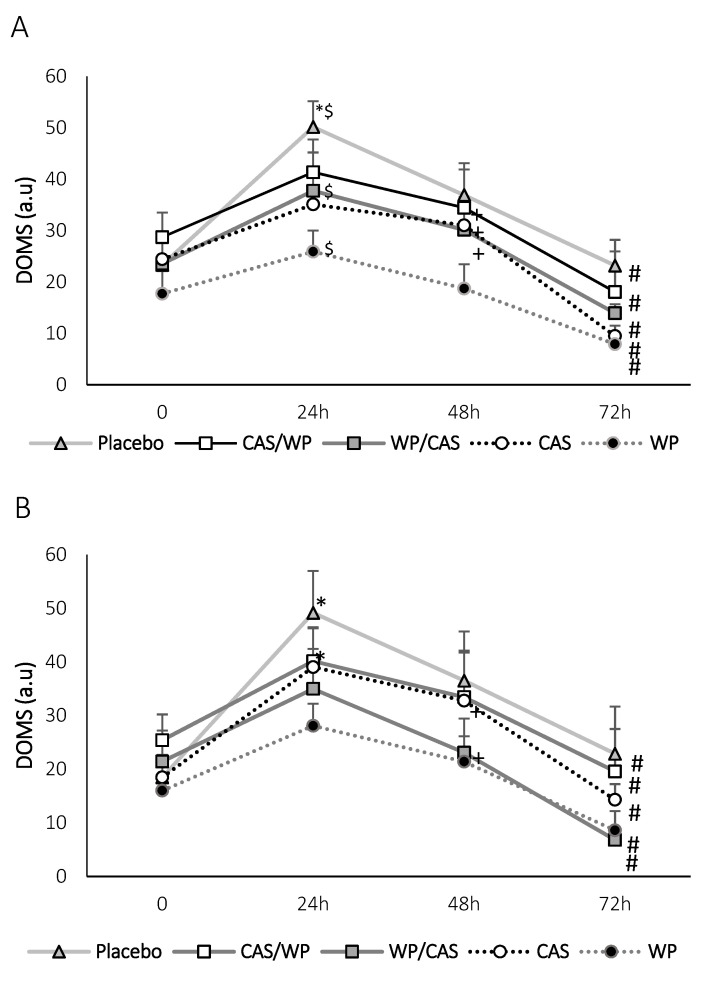
Delayed Onset Muscle Soreness (DOMS) post-treatments. (**A**) Down the stairs and (**B**) sitting. 0 immediately, 24 h, 48 h, and 72 h after the exercise protocol. a.u: Arbitrary Units. Values expressed as the mean ± SEM. * Significant differences between 0 h and 24 h, $ significant differences between PLA–WP/CAS and PLA–WP, # significant differences between 24 h and 72 h, and + significant differences between 48 h and 72 h. Significance level considered *p* < 0.05. *n* = 10. WP: Whey protein, CAS: casein. WP/CAS: 80% whey protein/20% casein. CAS/WP: 80% casein/20% whey protein. PLA: Placebo.

Furthermore, the results of the DOMS stairs and sitting assessments showed no interactions between the time and supplements (*p* = 0.414 and *p* = 0.665, respectively). The DOMS down the stairs assessment showed a significant increase only in the PLA treatment between 0 h and 24 h post-treatment (*p* = 0.002) (Figure 2A). Although not significant, the DOMS down the stairs also increased in the other groups at 24 h post-intervention. When comparing the 24-h and 48-h time points, a significant decrease was observed in CAS/WP, WP/CAS, and CAS (*p* = 0.031, *p* = 0.029 and *p* = 0.002, respectively). As for the DOMS sitting test, a significant increase between the 0-h and 24-h post-exercise sessions in the PLA and CAS treatments (*p* < 0.001), as well as the analyzed DOMS between the 24-h and 72-h post-exercise sessions, a significant decrease was found in all treatments (*p* < 0.01) (Figure 2B). When comparing the times between the 24-h and 48-h post-exercise sessions, a significant decrease was observed in the WP/CAS and CAS treatments (*p* = 0.025 and *p* = 0.008, respectively). All groups showed lower DOMS for down the stairs and sitting tests at 72 h post-intervention when compared to 24 h. These data demonstrate, although a single dose of WP, WP/CAS, and CAS/WP increased the amino acid concentration, that a longer period of permanent amino acids did not influence the performance but minimized muscle soreness.

The effects of the supplements in the markers on protein metabolism were assessed by the quantification of creatinine and urea blood levels, as well as urinary nitrogen excretion, and calculated the nitrogen balance considering nitrogen excretion and protein intake. Table 4 shows no significant differences regarding the protein markers analyzed, and the nitrogen balance was adequate in all the groups. 

Finally, the nutritional assessments before (PRE) and 24 h after the exercise and blood collection protocol (POST) are shown in Table 4. There was no interaction between group and time in terms of energy intake (Kcal), protein, fat, and carbohydrate intake, and the intakes were similar among the groups, which ensured that there was no impact from food intake in the results found. Additionally, when analyzing specifically the intake of proteins, on average, the participants consumed 1.49 g/kg/day at PRE and 1.70 g/kg/day at POST. These values represented the participants’ food consumption regardless of the type of supplement used and reached the protein intake requirements for healthy males [4].

## 4. Discussion

Given the importance of protein intake and amino acids availability for muscle anabolism, in the current study we compared the effects of whey protein and casein supplementation on the plasma amino acids and final metabolites of protein metabolism concentrations in physically active individuals. The main findings were: (1) the peak of BCAA occurred at the same time (60 min post ingestion) for WP, WP/CAS, and CAS/WP; (2) WP/CAS and CAS/WP blends promoted higher amino acid concentrations, and it was similar to the WP treatment when compared to CAS and PLA treatments; (3) Leucine and isoleucine presented a longer period of permanence in the WP, WP/CAS, and CAS/WP treatments; (4) CAS treatment promoted the peak of BCAA later at 120 min post-treatment, and after that, in the 180-min time point, the concentrations decreased; and (5) although a single dose of WP, WP/CAS, and CAS/WP increased the amino acid concentration, the longer period of permanence amino acids did not influence the performance but minimized the muscle soreness compared to CAS and PLA.

A rich amino acid environment is essential to activate different pathways like mTOR and can improve the anabolic muscle mass process [27,47]; therefore, we were expecting that the use of a blend of proteins in a similar composition of human breast milk (whey protein:casein) would offer an advantage over a single protein supplement on the plasma amino acid concentrations and DOMS recovery; all findings should be interpreted within this context. However, an advantage in the association of whey protein and casein (WP80%/CAS20%, similar to human breast milk) was not observed to increase the levels of the circulating amino acids when compared to the isolated WP or CAS treatments for a period greater than 120 min post-intervention. However, the concentrations of isoleucine and leucine in the WP/CAS and CAS/WP treatments were significantly higher than CAS and PLA treatments.

It is important to consider that the “window of opportunity” for muscle mass synthesis occurs in the 2 h post-exercise [48]. Interestingly, the higher concentrations of the BCAA were maintained for 120 min post treatment, which reaches the “window of opportunity” timing. Exercise is the principal factor to stimulate intramuscular signaling events that regulate mTOR pathway and skeletal muscle hypertrophy [49], and the increased amino acids availability post exercise provides substrate for protein synthesis initiated by exercise. However, 50% of the available amino acids from the ingested protein meal will be used for the gut energy production and for local protein synthesis [50]. The other ~50% of amino acids will be taken up by the liver and, from this only ~2.2 g or 11% of the amino acids (considering a dose of 20 g of protein supplement) is used for protein synthesis [51]. The remaining amino acids are catabolized and used as substrates for energy production, urea synthesis and other metabolic processes [49]. Although we observed a longer period of permanence for leucine and isoleucine post WP, WP/CAS, and WP/CAS intake, we applied an acute intervention, and there is no guarantee that the amino acids provided will be used for MPS. Furthermore, there is no consensus on the effect of amino acids intake immediately post-exercise optimization to muscle synthesis and recovery.

Moreover, the participants were classified as adults, and non-obese people, all reported familiarization and physical training practice at least three times a week at a moderate intensity. Some studies verify performance with age and suggest that physical exercise is essential for maintaining physical function and physiological health throughout life [52]. When analyzed the maximal muscle strength—one repetition maximum 1RM we observe an increase through session in all participants since 275 ± 42 to 339 ± 46 kg/1RM we observe and the participants relate that each resistance session was from moderate to high intensity when protocol was executed between 80–85% of this weight, because some participants could not maintain the same intensity during series, independent of age. Important to resalting that the amino acid concentration could be altered by the body mass index, metabolic disease, and a small number of participants could influence, but interestingly, the response to each supplement was similar between the times, as shown in Supplementary Figure S1.

Additionally, it was expected that protein supplementation could improve parameters related to the DOMS [53,54,55]. According to Ra et al. [41], DOMS in response to muscle stimuli is influenced by feeding or fasting before exercise performance. We showed that the protein supplementation associated with exercise protocol, followed by food intake, reduced the subjective perception of pain in the WP, CAS, WP/CAS, and CAS/WP groups when compared to the placebo treatment. However, in our study significant differences were observed in DOMS when comparing the times 0 h, 24 h, 48 h, and 72 h for stairs and sitting assessments after the exercise and placebo or protein supplementation protocols. We showed that only PLA and CAS treatments increased DOMS for both protocols between 0 h and 24 h post treatment and that was a protective effect to muscle soreness in the 24 h and 48 h time points in the CAS/WP, WP/CAS, and CAS treatments. These data demonstrated that, although a single dose of WP, WP/CAS, and CAS/WP increased amino acids concentration, the longer period of permanence amino acids did not influence performance but minimized muscle soreness. When analyzing the training effect by RPE, DOMS, we could observe lower pain perception in the fifth session (21 ± 32 a.u.) compared with the first session (40 ± 08 a.u.) when evaluated the mean of down the stairs pain perception, respectively. It is important to report that these results occurred independently of the protein supplemented.

Controversies still exist regarding the combination of whey protein and casein and the ideal ratio to stimulate the maintenance of circulating amino acids; it is well-established that sufficient protein intake and regular exercise training should form the cornerstone of any skeletal muscle hypertrophy diet. Even though the protein dose [56,57], source [58,59], and timing [60] may optimize skeletal muscle hypertrophy, the daily protein intake of ~1.6 g/kg/day obtained from supplements or high-quality protein sources at each meal throughout the day seems to be the most important factor to consider when increasing muscle mass with resistance exercise is the goal. 

Our study is the first to investigate the amino acids peak and permanence after a single bout of fasting and resistance exercise session followed by protein or placebo supplementation, specifically considering the proportion of WP and CAS of the human breast milk. The main point of this investigation is its randomized crossover design, which is essential for minimizing bias. Although the PLA treatment (composed by maltodextrin) in this study can be an adequate control group, the lack of a pure placebo condition (free of any macronutrient) may be considered a limitation; additionally, the small sample size limits the statistical power and generalizability. Another limitation would be the determination on the total plasma amino acids, which could enrich the manuscript results also help to understand nitrogen metabolism.

Moreover, it is important to consider that the concentrations of BCAA also depend on other nonevaluated factors, such as food intake in the long term and gut absorption capacity, which may have affected the results.

## 5. Conclusions

We concluded that, despite no positive effects observed in the protein metabolism markers, the amino acid kinetics highlighted that the WP seems to be the most effective supplement to increase the leucine concentration in plasma, and there was no advantage in the association of WP and CAS in the amino acids peak and period of permanence when compared to WP itself, but it minimized muscle soreness compared to CAS and PLA. Altogether, our data suggest that the WP, WP/CAS, and CAS/WP treatments promoted a similar positive effect in skeletal muscle recovery. Future investigations should be carried out in a larger sample and evaluate the WP and CAS blend supplementation impact exercise recovery and post-exercise BCAA profile in a long-term intervention.

## Figures and Tables

**Table 1:** Amino acid composition in 100 g of each protein supplement (g/100 g).

Amino Acid	WP	CAS	WP/CAS	CAS/WP
Aspartic Acid	8.66	5.19	7.97	5.88
Glutamic Acid	13.91	10.03	13.13	10.81
Alanine	3.89	7.83	4.68	7.04
Arginine	2.24	8	3.39	6.85
Cystine	1.46	0	1.17	0.29
Phenylalanine	2.63	1.76	2.46	1.93
Glycine	1.56	15.39	4.33	12.62
Histidine	1.56	0.7	1.39	0.87
Isoleucine	4.77	1.14	4.04	1.87
Leucine	8.76	2.73	7.55	3.94
Lysine	7.49	3.69	6.73	4.45
Methionine	2.92	0.79	2.49	1.22
Proline	1.56	11.79	3.61	9.74
Serine	2.82	2.81	2.82	2.81
Tyrosine	2.34	0.18	1.91	0.61
Threonine	5.64	1.41	4.79	2.26
Tryptophan	1.26	0	1.01	0.25
Valine	4.67	2.11	4.16	2.62

Notes: WP: 100% whey protein; CAS: 100% casein; WP/CAS: blend of 80% whey protein and 20% casein; CAS/WP: blend of 80% casein and 20% whey protein.

**Table 2:** Branched-chain amino acid concentration (µmol/L), differences between the supplements and time points.

Groups	Amino Acid	Rest	0 min	60 min	120 min	180 min	240 min	300 min
WP	Valine	270 ± 34	287 ± 35	423 ± 25.5 ^a,b^	379 ± 23 ^a,b^	297 ± 32 ^c,d^	266 ± 39 ^c,d^	272 ± 35 ^c,d^
	Isoleucine	28 ± 4	29 ± 4	67 ± 9 ^a,b^	49 ± 5 ^a,b,c^	33 ± 5 ^c,d^	28 ± 4 ^c,d^	27 ± 4 ^c,d^
	Leucine	61 ± 12	64 ± 12	150 ± 9 ^a,b^	116 ± 10 ^a,b,c^	79 ± 12 ^c,d^	62 ± 12 ^c,d^	65 ± 13 ^c,d^
CAS	Valine	340 ± 47	314 ± 41	405 ± 32 ^b^	417 ± 28 ^b^	370 ± 37	331 ± 44	373 ± 30
	Isoleucine	37 ± 5	31 ± 45	48 ± 5 ^b^	50 ± 4 ^a,b^	41 ± 6	33 ± 6 ^d^	36 ± 5 ^d^
	Leucine	83 ± 14	72 ± 13	114 ± 9 ^a,b^	120 ± 7 ^a,b^	101 ± 14	77 ± 14 ^c,d^	93 ± 10
WP/CAS	Valine	352 ± 28	330.5 ± 24	449 ± 25 ^a,b^	391 ± 34	384 ± 27	399 ± 36	360 ± 25
	Isoleucine	35 ± 4	31 ± 3	72 ± 6 ^a,b^	57 ± 6 ^a,b,c^	43 ± 6 ^c,d^	41 ± 5 ^c,d^	37 ± 3 ^c,d^
	Leucine	80 ± 9	70 ± 8	138 ± 9 ^a,b^	118 ± 8 ^a,b^	98 ± 9 ^c^	96 ± 10 ^c^	90 ± 8 ^c^
CAS/WP	Valine	351 ± 34	303 ± 33	424 ± 30 ^b^	412 ± 27 ^b^	341 ± 42	336 ± 27 ^c^	346 ± 26
	Isoleucine	36 ± 4	28 ± 4	67 ± 5 ^a,b^	48 ± 4 ^a,b,c^	34 ± 6 ^c,d^	33 ± 3 ^c,d^	31 ± 3 ^c,d^
	Leucine	86 ± 12	63 ± 11	136 ± 7 ^a,b^	114 ± 9 ^a,b,c^	81 ± 15 ^c,d^	72 ± 12 ^c,d^	80 ± 10 ^c,d^
PLA	Valine	326 ± 31	311 ± 26	300 ± 25	301 ± 26	321 ± 29	367 ± 16	333 ± 39
	Isoleucine	32 ± 3	27 ± 3	23 ± 2	24 ± 3	29 ± 4	36 ± 4	33 ± 5
	Leucine	76 ± 10	68 ± 8	48 ± 7 ^a^	48 ± 9 ^a^	64 ± 10	83 ± 8 ^c,d^	83 ± 11 ^c,d^

Data expressed as mean and mean standard error (mean ± SEM). Letters represent statistical differences between time points. ^a^ = vs. Rest; ^b^ = vs. 0 min; ^c^ = vs. 60 min; ^d^ = vs. 120 min. vs. = versus (*p* < 0.05) by ANOVA two-way repeated measures, e.g., if the letter “a” is in the times 60 min and 120 min, this represents that Rest is statistically different from 60 and 120 min. Rest: immediately before exercise. 0 min: immediately after exercise and supplementation. 30 min, 60 min, 120 min, 180 min, 240 min, and 300 min: minutes after exercise and supplementation. WP: Whey protein and CAS: casein. WP/CAS: 80% whey protein/20% casein. CAS/WP: 80% casein/20% whey protein. PLA: Placebo.

**Table 3:** Participant characteristics and exercise performances during each training session.

Physical Performance	WP	CAS	WP/CAS	CAS/WP	PLA
1RM (kg)	307.5 ± 44.9	306.9 ± 52.1	306.6 ± 47.8	309.8 ± 50.6	322.7 ± 50.4
Leg press (kg)	255.5 ± 43.2	256.48 ± 47.7	257.8 ± 44.9	264.3 ± 36.4	266.9 ± 39.9
RPE (a.u)	8.1 ± 1.2	8.2 ± 0.8	7.9 ± 1.2	8.1 ± 1.4	8.3 ± 1.4
H.R (bpm)	125.3 ± 29.05	131.1 ± 23.1	129.6 ± 20	123.7 ± 22	132.3 ± 18.4
TRIMP RPE (a.u)	2957 ± 685	3094 ± 544	3058 ± 36	2920 ± 520	3082 ± 428
TRIMP Vol (kg)	1010 ± 169	1005 ± 133	1012 ± 123	1046 ± 150	1054 ± 144
kcal	182.2 ± 9.9	181.7 ± 10.2	183.3 ± 9.9	180.8 ± 9.9	182.3 ± 9.6

Note: WP: Whey protein, CAS: casein. WP/CAS: 80% whey protein/20% casein. CAS/WP: 80% casein/20% whey protein. PLA: Placebo. 1RM (kg): 1 Maximum repetition test, Leg press (kg): exercise training load, and RPE: Rating of perceived exertion. a.u: arbitrary units. H.R: Heart rate. TRIMP RPE: Global training index (RPE × min). TRIMP Vol: Global training load index Volume session (kg). kcal: total energy spent in the exercise session. Values shown as mean ± SEM. *n* = 10.

**Table 4:** Protein metabolism markers post each treatment and the nutritional assessments pre and post the treatments.

**Protein Metabolism Markers**	**WP**			**CAS**			**WP/CAS**			**CAS/WP**			**PLA**		
Creatinine (mg/kg/24 h)	25.6 ± 2.8			17.2 ± 2.5			22.3 ± 2.5			25.3 ± 3.9			24 ± 3.1		
Urea (g/24 h)	27.3 ± 2.8			21.3 ± 3			24.9 ± 2.9			26.1 ± 2.9			23 ± 2.9		
UN (g/24 h)	15.3 ± 1.9			12.7 ± 1.7			13.5 ± 1.6			15 ± 2.1			11.6 ± 1.4		
NB (g/24 h)	2.6 ± 4.7			−0.1 ± 2.3			8 ± 2.5			2.4 ± 2.6			3.7 ± 2.7		
**Nutrients** **(Dietary record)**	**WP**			**CAS**			**WP/CAS**			**CAS/WP**			**PLA**		
	**PRE**	**POST**	**Δ%**	**PRE**	**POST**	**Δ%**	**PRE**	**POST**	**Δ%**	**PRE**	**POST**	**Δ%**	**PRE**	**POST**	**Δ%**
kcal	2567 ± 367	2181 ± 336	−17	2351 ± 257	1830 ± 255	−28	2185 ± 365	2360 ± 169	7	1964.5 ± 225	2323 ± 209	15	2271 ± 415	2303 ± 270	1
PTN (g)	150 ± 30	137 ± 31	−9	115 ± 11	103 ± 13	−11	108 ± 15	166 ± 18	35	96.1 ± 13	133 ± 14	28	103 ± 22	115 ± 14	10
FAT (g)	89 ± 19	70 ± 14	−25	77 ± 9	68 ± 8	−13	90 ± 26	85 ± 7	−6	59.2 ± 6	89 ± 14	33	98 ± 34	77 ± 13	−27
CHO (g)	290 ± 33	274 ± 39	−5	267 ± 35	224 ± 35	−19	231 ± 41	256 ± 23	10	261.7 ± 42	251 ± 30	−4	243 ± 40	436 ± 161	44

Note: PRE: Food register 24 h before exercise and supplementation protocol. POST: Food register 24 h after exercise and supplementation protocol. NB: nitrogen balance. UN: urinary nitrogen. WP: Whey protein, CAS: casein. WP/CAS: 80% whey protein/20% casein. CAS/WP: 80% casein/20% whey protein. PLA: Placebo. Δ%: Delta of variation between PRE and POST. Values shown as the mean ± SEM. *n* = 10.

## Data Availability

Data can be made available from the corresponding author upon reasonable request.

## Supplementary Materials

The following are available online at https://www.mdpi.com/article/10.3390/nu13072153/s1: Figure S1: Plasma concentration of branched-chain amino acids (Valine, Isoleucine and Leucine) for each treatment.
