# Supplementary material for: Casein and Whey Protein in the Breast Milk Ratio: Could It Promote Protein Metabolism Enhancement in Physically Active Adults?
Source: Nutrients. 2021 Jun 23;13(7):2153. doi: 10.3390/nu13072153 (PMC8308344; doi:10.3390/nu13072153)
Supplement: Supplementary file 1 [file nutrients-13-02153-s001.zip › nutrients-1217779-supplementary.pdf]

Supplementary Figure

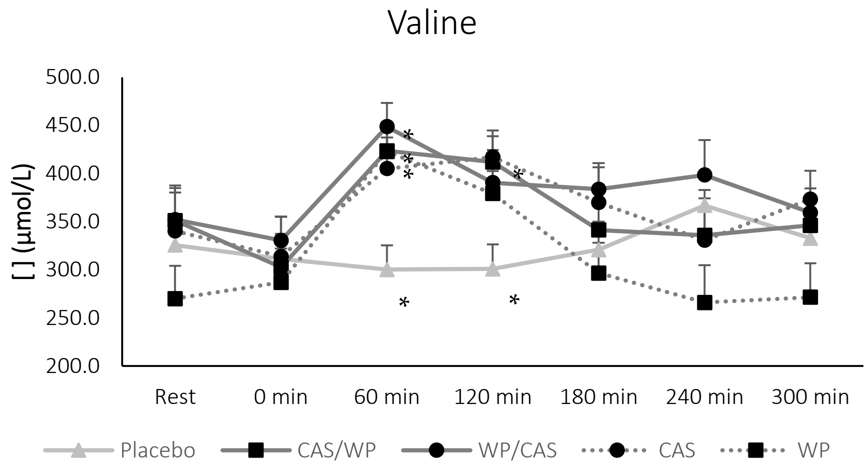

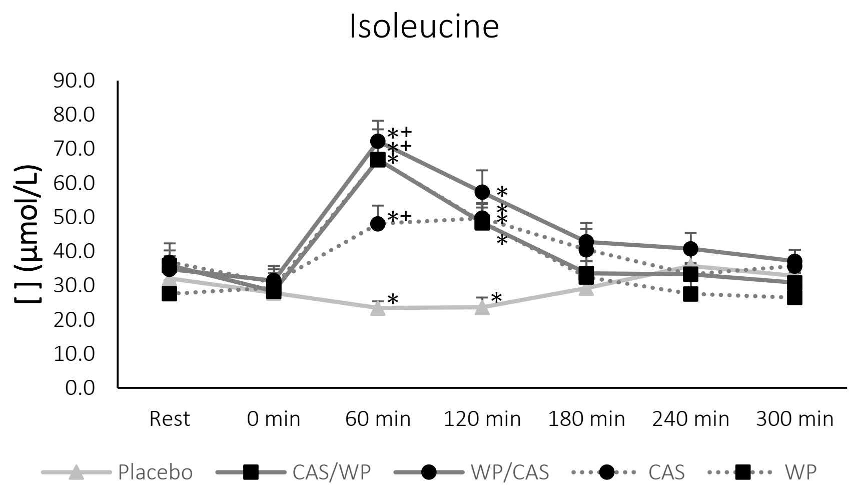

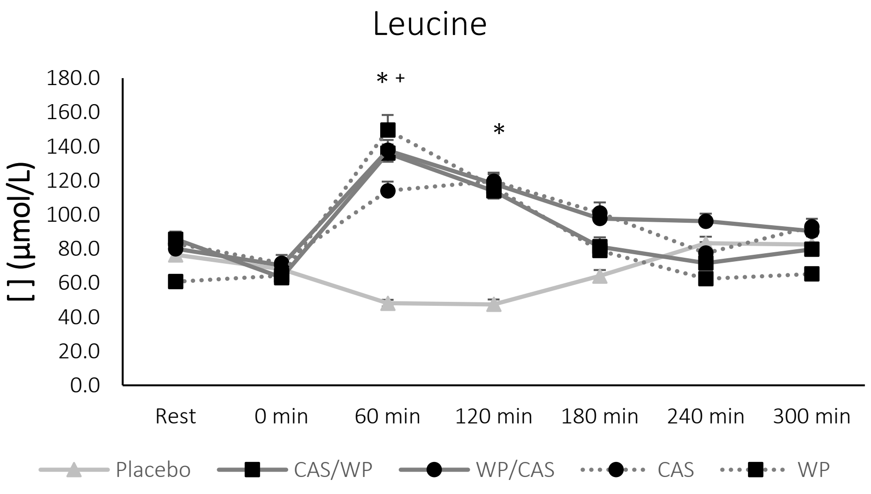

Figure S1. Plasma concentration of branched-chain amino acids (Valine, Isoleucine and Leucine) for each treatment. Concentration of plasma amino acids at different times before (Rest), and after experimental protocol (0 min, 60 min, 120 min, 180 min, 240 min, 300 min). Values expressed in Mean ±SEM. significance level at $p < 0.05$. *Significant difference between Placebo and other supplements the 60 and 120 min, $^{+}$ Significant difference between CAS and other supplements. CAS/WP: 80% casein/20% whey protein; PLA: Placebo, WP/CAS: 80% whey protein/20% casein, CAS: casein, WP: Whey protein. n = 10.
